# A thematic analysis of what Australians state would change their minds on climate change

**DOI:** 10.1038/s41598-025-96714-z

**Published:** 2025-04-22

**Authors:** Amy S. G. Lee, Kelly Kirkland, Samantha K. Stanley, Abby Robinson, Zoe Leviston, Iain Walker

**Affiliations:** 1 Melbourne School of Psychological Sciences, University of Melbourne, Melbourne, Australia; 2 School of Psychology, University of Queensland, Brisbane, Australia; 3 UNSW Institute for Climate Risk and Response, University of New South Wales, Sydney, Australia; 4 School of Psychology, University of New South Wales, Sydney, Australia; 5 School of Arts and Humanities, Edith Cowan University, Joondalup, Australia; 6 School of Medicine and Psychology, The Australian National University, Canberra, Australia

**Keywords:** Climate change, Audience segmentation, Six Americas, Large language models, Chat GPT, Opinion change, Psychology, Human behaviour

## Abstract

What do Australians believe would change their current opinions about climate change? In this study, we used audience segmentation analysis through the Six Americas Short Survey to identify groups of climate opinion holders within a representative sample of Australians. We had 4857 participants tell us what it would take to change their current opinions about climate change and leveraged OpenAI’s Generative Pre-Trained Transformer (GPT) to identify the presence or absence of themes (*Nothing*, *Evidence and Information*, *Trusted Sources*, *Action*, and *Unsure*) and subthemes in their responses. GPT performed at near-human levels, proving to be a highly useful tool for thematic analysis. Our analyses revealed that strong climate denialists and believers tended to display greater dogmatism, with increased likelihood of stating that nothing would change their mind and lower likelihood of being unsure. Results also highlighted the need for diverse forms of evidence and information and the importance of trusted sources of information across audience segments. These findings provide support for GPT’s utility in managing large datasets in the social sciences and offer participant-informed insights into climate opinion change.

**Supplementary Information:**

The online version contains supplementary material available at 10.1038/s41598-025-96714-z.

## A thematic analysis of what Australians state would change their minds on climate change

Climate change poses an existential threat to societies and ecosystems^1^. Responding to this threat demands unprecedented changes to global systems^2^, dependent, in part, on people’s beliefs about climate change and its risks^3–5^. Despite well-established scientific consensus about the existence, origins, and consequences of climate change^6^, a substantial minority remain unconcerned about its risks or unconvinced about its existence^7^. Others may be more concerned, yet susceptible to (dis- or mis-) information that could shift them towards skepticism or prevent concern translating into action. Understanding what it would take to change people’s current beliefs about climate change is therefore a critical step in garnering and maintaining support for global climate reform.

Climate change opinions are trending towards increased concern^8,9^. While public opinion snapshots indicate relatively modest shifts, longitudinal panel data highlights considerable volatility in individuals’ views. In Australia, for instance, 48.5% of participants changed their opinion about climate change at least once in 5 years^10^. Acceptance of human-induced climate change is therefore not necessarily an “endpoint”, but an attitudinal position subject to change. There is a strong and growing literature on interventions that affect climate-related beliefs and behaviors, such as learning about scientific consensus^11,12^, exposure to framed information^13^, and inoculation against climate misinformation^14^. However, no research to our knowledge has asked people what factors *they think* would change their beliefs.

Some research offers hints about the factors to which people attribute their current positions. For example, interviews with young Australians found many attributed their understanding of climate change to social and news media in the absence of coverage in the curriculum^15^, suggesting a larger perceived role of peer influence and journalism than formal education. Further clues might be gleaned by examining (dis)trusted sources of climate information. In a study spanning eight countries, Ejaz and colleagues^16^ found that belief in climate misinformation was associated with lower trust in scientists, environmental activists, and international organizations, and higher trust in celebrities, politicians, and energy companies. Most relevant to our work, Leviston and colleagues^17^ asked people to state the basis of their climate change opinions, selecting from a short-list of themes drawn from open-ended responses to the same question in a previous survey. “Scientific research”, followed by “common sense”, and “the weather” were most frequently selected. Importantly, those who denied climate change more often selected “common sense”, while those convinced of anthropogenic climate change favored “scientific research”, and those who were unsure most often selected “the weather”.

Audience segmentation approaches acknowledge that the public are attitudinally diverse and define “segments” with similar views^8^. These approaches have been used globally^18^ and enable tailored engagement that responds to each segment’s current opinions^19^. The Six Americas approach has been used for decades to successfully profile Americans (and recently, Australians)^9,20^ according to their investment in their climate change views (i.e., issue involvement), and acceptance or rejection of the issue (i.e., attitudinal valence)^19^. By asking people to self-report how much they are worried about climate change, believe it will harm them personally and harm future generations, and believe the issue is important, we can determine which of six audience segments they belong to^21^: *Alarmed*,* Concerned*,* Cautious*,* Disengaged*,* Doubtful*, and *Dismissive* (from most-to-least convinced of climate risks). Issue involvement follows a U-shaped pattern across segments, with the *Alarmed* and *Dismissive* most invested in their (opposing) views, and thus more resistant to opinion change.

Neumann and colleagues^9^ documented a trend showing Australians were increasingly *Alarmed* about climate change in 2020 relative to 2016 and 2011. This trend appeared to be partially driven by upward movement from the less engaged, though accepting, segments: *Concerned* and *Cautious* memberships dropped in the same period, while *Doubtful* and *Dismissive* memberships grew slightly^22^. Those accepting climate change have perhaps become more invested in their stance, yet some *Cautious* and *Concerned* may have shifted towards skepticism. Meanwhile, the minority invested in doubting or denying climate change have remained stubbornly so, hindering effective engagement^23–25^. It is unclear what factors – if any – may change their views. Likewise, it is unclear what would prompt middle segments (*Concerned* to *Doubtful*) to revise their opinions towards either extreme, and whether the *Alarmed* view their opinions as crystalized and unchanging or if there are conditions that may prompt opinion shifts.

The digital era offers new opportunities to address these research gaps. Social scientists now frequently access large-scale data from nationally representative datasets^9,26^, multiple countries^27,28^, and online platforms^29,30^, which can yield valuable insights. However, traditional analysis methods are often labor-intensive, time-consuming, and require advanced skills^30^. Artificial Intelligence (AI), particularly Large Language Models (LMMs), offers scalable, cost-effective solutions to overcome these challenges^31^.

LMMs such as OpenAI’s Generative Pre-Trained Transformer (GPT), offer significant promise for managing qualitative data^31^. Relying on human coders^32^ is a valuable but expensive and labor-intensive approach that becomes impractical for large datasets. Automated text analysis tools, such as dictionary methods for word counts^29,33^ or sentiment analysis^34^, provide faster alternatives but often trade accuracy for simplicity, limiting their use to large, noisy datasets. Machine-learning techniques offer greater accuracy^30^ but typically demand substantial IT resources and coding expertise. GPT, by contrast, is a cost-effective and scalable alternative, capable of performing numerous tasks at human or near-human levels^35,36^. Rathje and colleagues^31^ found that GPT outperforms dictionary-based analyses for identifying sentiment, discrete emotions, and offensiveness in multiple languages, correlating at ~ 0.7 with human coders, compared to alternative dictionary-based methods which correlated at ~ 0.25. GPT is already being used for thematic analysis, including by Australia’s Commonwealth Scientific and Industrial Research Organisation (CSIRO)^37^. Thus, GPT represents a potentially valuable tool for analyzing large qualitative datasets, offering new opportunities to glean participant-informed insights.

We aimed to understand similarities and differences in how those holding different climate opinions describe what would change their views on climate change. We used the Six Americas Short Survey (SASSY)^21^ to identify six audience segments in a representative sample of Australians. Participants were asked to contemplate their opinions on climate change (e.g., whether they accept climate change and its causes), and provide open-ended responses on what might change their mind. Two independent human coders identified and negotiated a codebook featuring six dominant themes. Critically, GPT Application Programming Interface (API) was used to assess the presence and absence of themes across responses^31^. We established intercoder reliability between GPT and the human coders to validate its reliability for thematic analysis. We then assessed theme prevalence across SASSY segments and compared how they manifested by analyzing the prevalence of key subthemes. This project addressed the following research questions (RQ): (1) Which factors do climate change audience segments report would change their minds about climate change? (2) Is OpenAI’s GPT a reliable method for conducting robust thematic analyses of text-based data? The project was data-driven and exploratory, and we thus had no pre-established hypotheses.

## Results

To identify participants’ audience segments, we ran responses to the four SASSY items through Chryst et al.’s online tool^21^ (https://climatecommunication.yale.edu/visualizations-data/sassy). As reported in^9^, of the 5104 participants who could be segmented, 24.9% were *Alarmed*, 27.6% were *Concerned*, 23.0% were *Cautious*, 3.1% were *Disengaged*, 14.1% were *Doubtful*, and 7.3% were *Dismissive*.

### RQ 1: thematic analyses across SASSY segments

**Fig. 1:**
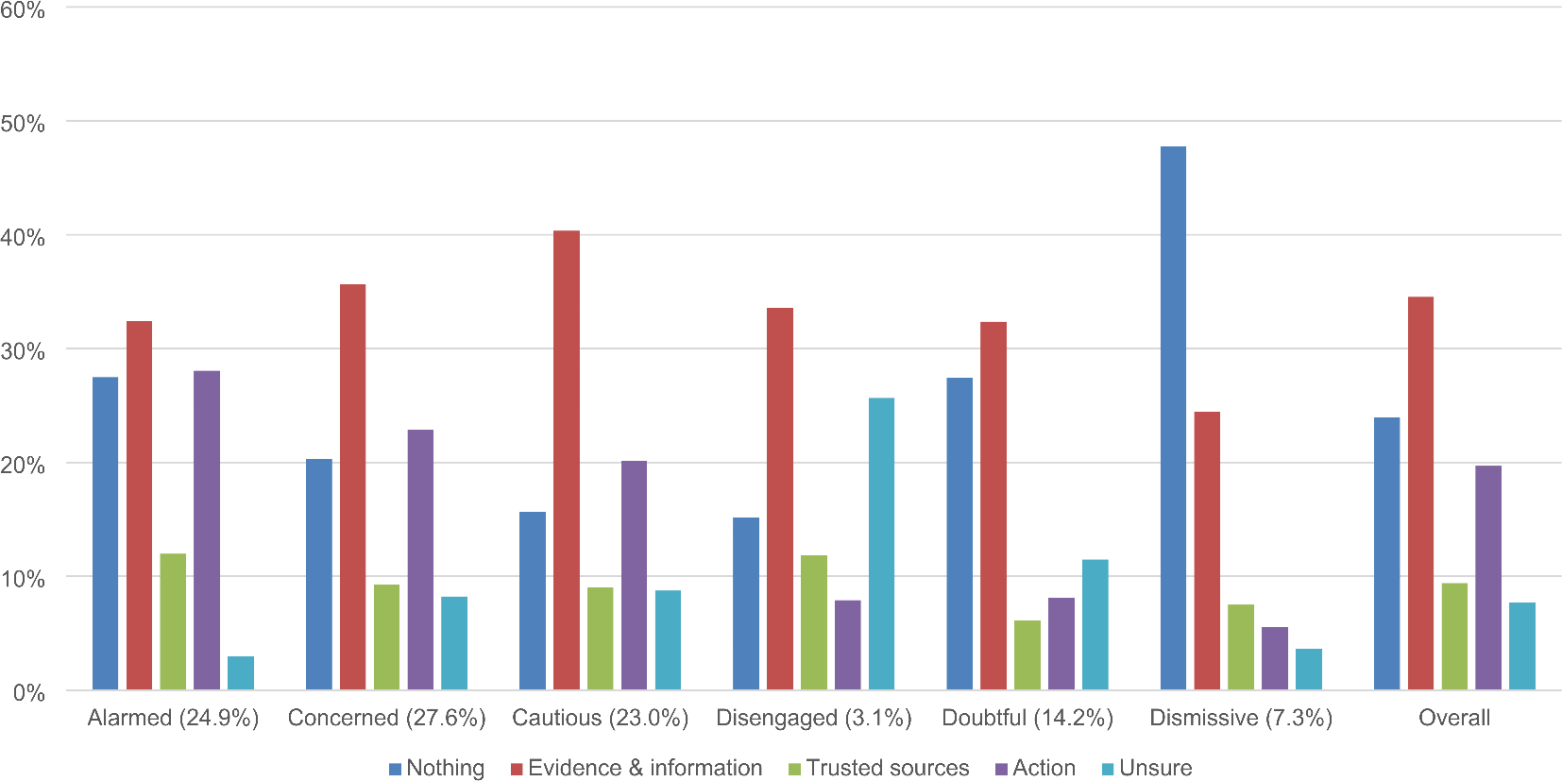
Standardized proportion of major themes across responses from each SASSY segment. *Note* Details for irrelevant responses coded to N/A are presented in the Supplementary Materials (Fig. S1).

Through inductive content analysis, two independent human coders identified six dominant themes in a random sample of open-ended responses (see Table 1 and Supplementary Table S1). GPT then identified the presence or absence of themes across responses, assigning multiple themes when necessary (see Supplementary Table S2 for theme co-occurrence). Of the 4857 participants who responded to the open-text question, GPT categorized 4849 responses to at least one theme, including a “not applicable” (N/A) theme for off-topic responses (see Supplementary Tables S3–S4 and Fig. S1). Once responses were thematically coded, we conducted Chi-square tests to examine how frequently themes were observed across SASSY segments. We separately analyzed each theme because coding was not mutually exclusive. In Fig. 1 and Table 2, we present the percentage of theme present across segments, so these are comparable across the differently-sized groups.

**Table 1:** Codebook of dominant themes.

Themes	Subthemes
1. **Nothing**: This theme applies to respondents who express a strong commitment to their current views and state that nothing would change their mind.	Not applicable
2. **Evidence and Information**: This theme applies to respondents who state that more evidence, information, and/or education would change their mind. This theme is concerned with the *content* of information.	2.1. **Scientific evidence**: Respondent states a desire or need for scientific evidence or research. 2.2. **Anthropogenic climate change**: Respondent describes a need for evidence that climate change is or is not caused by humans. 2.3. **Observable/experiential evidence or personally feeling climate impacts**: Respondent describes personally experiencing or observing that climate change is or is not occurring. 2.4. **Unbiased or transparent information**: Respondent describes a desire for more transparent information or less biased information, suggesting a hidden agenda in existing evidence and information. 2.5. **Simple/clearer information or education**: Respondent describes a need for clearer or simpler information or explanations about climate change. Alternatively, respondent expresses a desire for more education. 2.6. **Legitimate information**: Respondent expresses a desire for better, proper, or more legitimate information, suggesting doubt about the quality of existing evidence and information. 2.7. **Definitive or irrefutable evidence**: Respondent expresses a desire for definitive, irrefutable, or absolute evidence/proof that the climate is or is not changing.
3. **Trusted Sources**: Respondents who fall under this theme state that trusted sources of information would change their mind. Participants who state that greater consensus would change their mind should also be categorized as requiring trusted sources. This theme is concerned with the *source* who provides information.	3.1. **Scientists & experts in the field**: Respondent describes opinions from scientific or environmental organizations, scientists, or experts in the field. 3.2. **Consensus**: Respondent comments on a need for greater consensus (e.g., scientific/expert consensus, public consensus, political consensus, consensus between different groups). 3.3. **Unbiased sources of information or no vested interests**: Respondents describe a need for unbiased sources of information or sources of information that do not have vested interests. 3.4. **Media**: Respondent comments on the role of the media. Comments could include expressions of distrust towards the media or a desire for more media coverage or news reports. 3.5. **Government**: Respondent comments on the government or on government bodies. Comments could include desire for information from government/government bodies or distrust towards government/government bodies. 3.6. **Distrust towards existing information sources**: Respondent expresses distrust for existing information produced by individuals and public or private institutions. These institutions can include scientists and scientific organizations, the government, the media, politicians, and other public figures.
4. **Action**: Respondent states that actions taken by the government, corporations, or the public would change their mind. Respondents who state that a change in how people talk about or behave regarding climate change are also referring to greater action changing their minds.	4.1. **Action by government**: Respondent states that climate-related government actions (e.g., stronger climate policies) would change their mind about climate change. 4.2. **Actions by corporations and big business**: Respondent states that climate-related actions by corporations (e.g., stronger climate policies, pro-climate practices) would change their mind about climate change. 4.3. **Widespread individual behavior change**: Respondent states that actions by individuals, people, humans, or the general public could change their mind. 4.4. **Action on a global scale**: Respondent refers to actions by countries/governments around the world, beyond Australia. 4.5. **Depoliticization, deradicalization**: Respondent mentions a need for reduced politicization, radicalism, or extremism. Alternatively, respondent mentions a desire for increased level-headedness and rational debate between opposing parties.
5. **Unsure**: Respondent states they are unsure what would change their mind.	Not applicable
6. **N/A**: Respondent goes off on tangents that do not directly address the question: “What would change your mind about climate change?”.	Not applicable

Definitions have been shortened to streamline the table. Complete definitions fed to GPT are available in the Supplementary Materials.

All Chi-square tests were significant, indicating associations between SASSY segments and each theme. Post-hoc testing (see bolding and italicization in Table 2 and Supplementary Table S5) showed that *Alarmed* and *Dismissive* segments were more likely to respond that *Nothing* would change their minds about climate change, and less likely to be *Unsure*. The *Cautious* and *Concerned* were less likely to say *Nothing* would change their mind, and the *Disengaged* and *Doubtful* were more likely to be *Unsure*. *Cautious* participants were more likely, and *Dismissives* less likely, to say *Evidence and Information* would change their mind. *Trusted Sources* was more often cited by *Alarmed* participants and less so by *Doubtful* participants. Meanwhile, *Action* responses differed by attitudinal valence: the *Alarmed* and *Concerned* were more likely to say that *Action* would change their mind; the *Disengaged*,* Doubtful*, and *Dismissive* were less likely to give this response.

**Table 2:** Contingency table for the presence and absence of themes by SASSY profile.

	Nothing	Evidence & information	Trusted sources	Action	Unsure
Alarmed	**27.5**	32.4	**12.0**	**28.1**	*3.0*
Concerned	*20.3*	35.7	9.3	**22.9**	8.2
Cautious	*15.7*	**40.3**	9.0	20.1	8.7
Disengaged	15.1	33.6	11.8	*7.9*	**25.7**
Doubtful	27.4	32.4	*6.1*	*8.1*	**11.5**
Dismissive	**47.8**	*24.4*	7.5	*5.6*	*3.6*
Chi-square test	χ^2^(5) = 182.81, *p* < .001, V = 0.19	χ^2^(5) = 37.32, *p* < .001, V = 0.09	χ^2^(5) = 21.16, *p* < .001, V = 0.07	χ^2^(5) = 179.54, *p* < .001, V = 0.19	χ^2^(5) = 132.34, *p* < .001, V = 0.17

*N* = 4844. V = Cramer’s V effect size. Bolded and italicized cells depict significant findings at the adjusted threshold *p*-value < 0.004. Bolded cells indicate overrepresented use of themes, while italicized cells indicate underrepresented use of themes.

Table S6 in the Supplementary Materials presents results from a series of binary logistic regression analyses that predict the presence of themes based on participant demographics. Though very small, effect sizes indicate some patterns, such as increased reference to *Evidence and Information* and *Trusted Sources* for those with higher education levels, and higher incidence of *Nothing* among older participants.

**Fig. 2:**
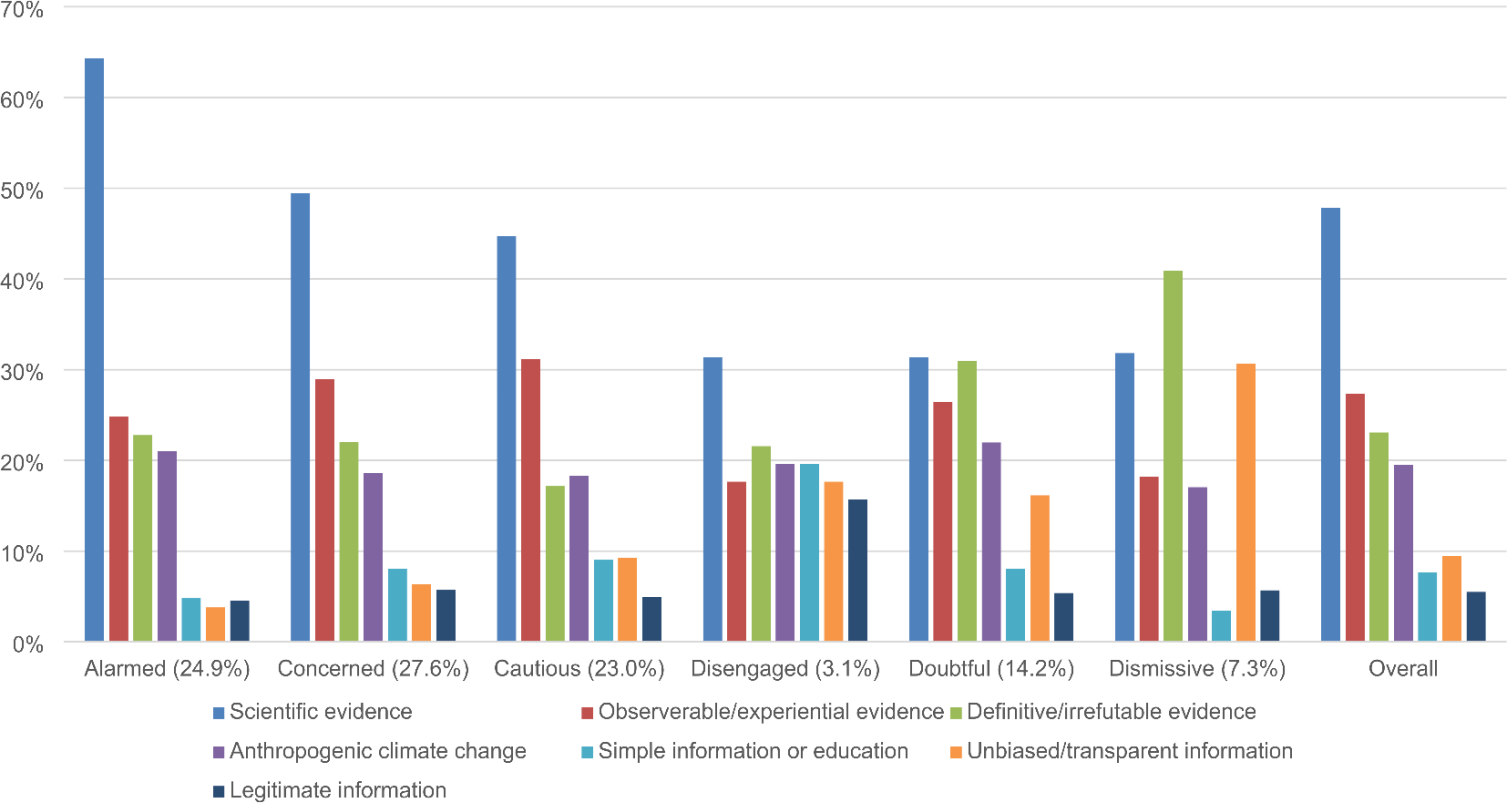
Standardized proportion of subthemes in responses coded to *Evidence and Information* from each SASSY segment.

**Fig. 3:**
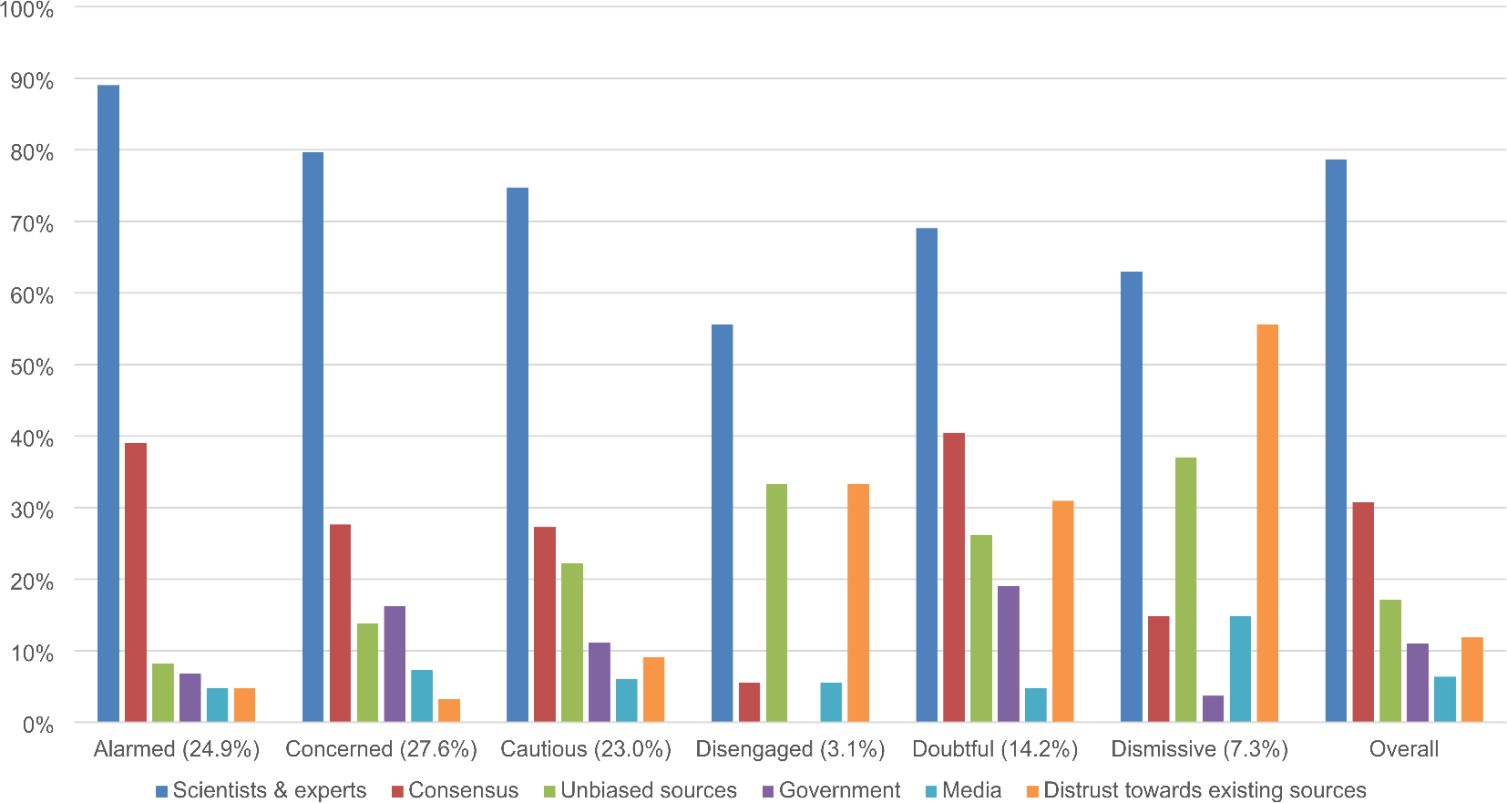
Standardized proportion of subthemes in responses coded to *Trusted Sources* from each SASSY segment.

**Fig. 4:**
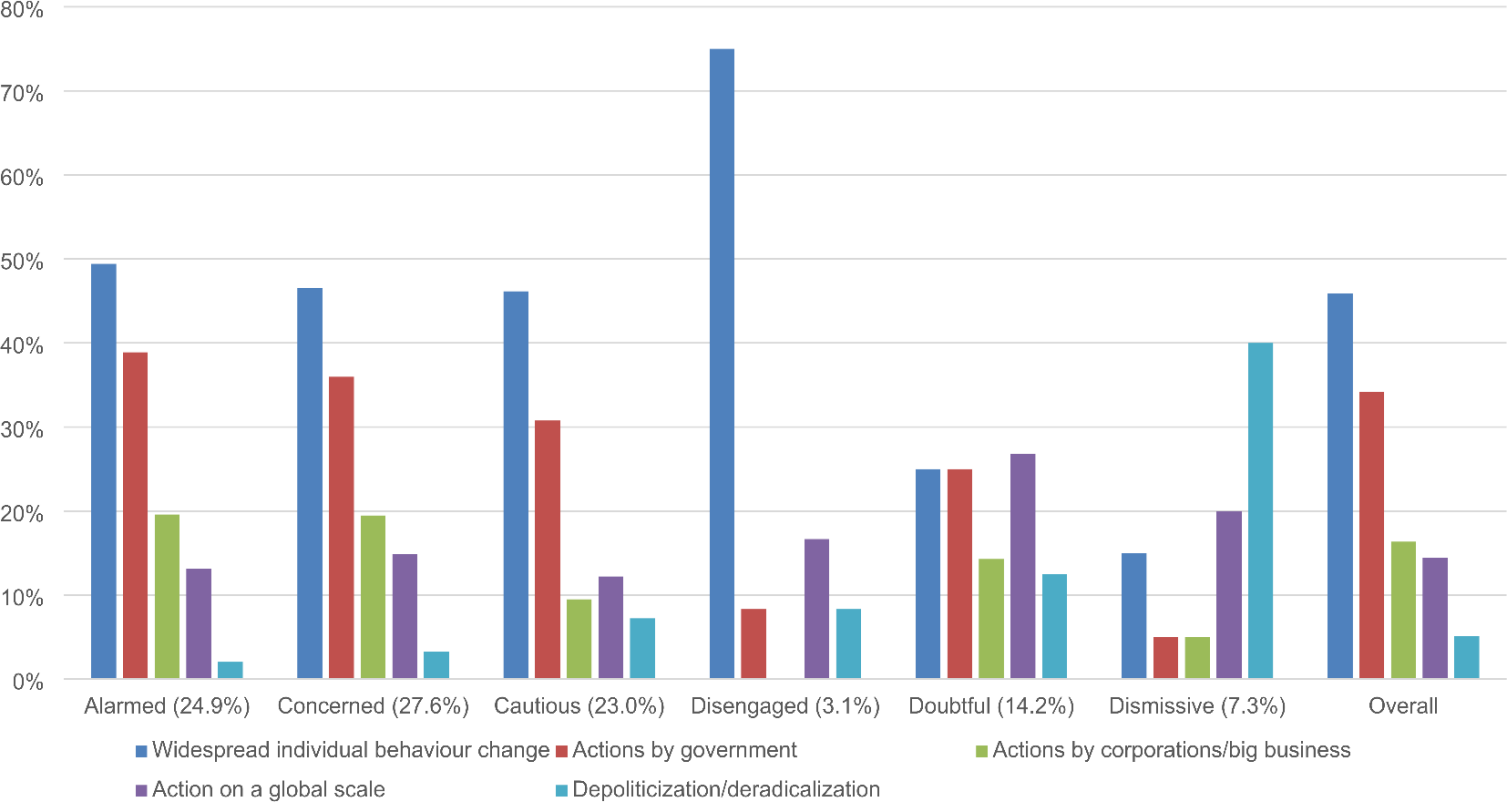
Standardized proportion of subthemes in responses coded to *Action* from each SASSY segment.

Further analyses were conducted to assess whether the *Evidence and Information*, *Trusted Sources*, and *Action* themes manifested differently across SASSY segments. We used the same process as above to identify and instruct GPT to code dominant subthemes (see Table 1). As subthemes were not mutually exclusive, GPT was permitted to assign responses to multiple subthemes when necessary. Figures 2, 3 and 4 present the prevalence of these subthemes as a percentage of all responses by each segment coded to *Evidence and Information*, *Trusted Sources*, and *Action*, respectively. We did not test for statistically significant differences because of insufficient power.

The prevalence of subthemes appeared to differ by attitudinal valence. Although many responses coded to *Evidence and Information* referenced scientific evidence, this subtheme was relatively more common across the *Alarmed*, *Concerned*, and *Cautious*. By contrast, the *Dismissive* and *Doubtful* were relatively more likely to reference definitive or irrefutable evidence and unbiased or transparent information, while the *Disengaged* cited simple information or education. Across SASSY segments, responses coded to *Trusted Sources* most often referred to scientists and experts. However, the *Disengaged*, *Doubtful*, and *Dismissive* also frequently expressed distrust towards existing information sources and a desire for unbiased sources. Responses coded to *Action* (except by the *Dismissive*) often described widespread individual behavior change and government actions. Additionally, depoliticization or deradicalization was a more important subtheme for participants holding skeptical views.

### RQ 2: intercoder reliability between GPT and human coders

We validated GPT’s capacity to thematically code responses by assessing intercoder reliability between GPT and two human coders. Using the thematic codebook, the human coders and GPT independently analyzed a random sample of responses. Given the uneven representation of themes, we analyzed intercoder reliability between the human coders and GPT for each theme and relied on Gwet’s AC(1) as the most appropriate measure^38^. Gwet’s chance-corrected approach^39^ compensated for the uneven representation of themes and small number of raters, allowing us to apply Landis and Koch’s intercoder reliability benchmarks^40^ to agreement coefficients.

Although intercoder reliability was higher between the two human coders than between each human coder and GPT, GPT still showed high levels of consistency. Table 3 shows “almost perfect agreement” between the human coders and GPT for each theme, per Landis and Koch’s benchmarks^40^. This level of agreement exceeds the threshold of intercoder reliability previously accepted in content analysis projects using human coders. For example, McDaniel and colleagues^41^ retained variables with an AC(1) value ≥ 0.60 or “moderate” agreement. Additionally, Abbas and colleagues^42^ concluded that “substantial” and “almost perfect” agreement between coders of qualitative interviews demonstrates content analysis “rigor and quality”. We therefore determined that GPT had achieved sufficient reliability to thematically code all responses.

**Table 3:** Summary statistics for intercoder reliability.

	Coder 1 & 2				Coder 1 & GPT				Coder 2 & GPT			
	Gwet’s AC(1)	95% CI		Percentage agreement	Gwet’s AC(1)	95% CI		Percentage agreement	Gwet’s AC(1)	95% CI		Percentage agreement
		LL	UL			LL	UL			LL	UL	
Nothing	0.96	0.94	0.99	98	0.96	0.93	0.98	97	0.95	0.93	0.98	97
Evidence & info	0.95	0.92	0.98	97	0.87	0.82	0.92	93	0.87	0.83	0.92	93
Trusted sources	0.97	0.95	0.99	97	0.94	0.91	0.97	95	0.95	0.93	0.97	96
Action	0.98	0.97	1.00	99	0.88	0.83	0.92	91	0.86	0.82	0.91	90
Unsure	0.99	0.98	1.00	99	0.98	0.97	1.00	99	0.98	0.96	1.00	98

* CI* confidence interval, *LL* lower limit, *UL* upper limit. All Gwet’s AC(1) values reflect “almost perfect agreement”^40^. Summary statistics for irrelevant responses coded to *N/A* are available in the Supplementary Materials (Table S4).

## Discussion

In 2023 and 2024, the world shattered numerous climate records^43^: global temperatures skyrocketed at an alarming rate, ocean temperatures increased beyond model predictions, and Antarctic ice volume dropped by unprecedented levels. The world’s climate is changing, yet beliefs about its risks vary substantially^9^. Here, we asked a representative sample of Australians what factors they think would change their mind on climate change and compared major theme and subtheme prevalence across SASSY segments. Analyses revealed important insights on what individuals report would change their mind on climate change, though the factors identified may not necessarily align with what changes minds in practice, as we discuss below. Responses showed dogmatism from participants with high issue involvement and the need for diverse forms of evidence and information and trusted sources of information. We also further validated GPT API’s capacity to identify the presence and absence of themes from qualitative responses^31^. This method proved to be a cost-effective and reliable way to draw meaning from vast amounts of qualitative data.

### RQ 1: what factors Australians believe would change their current stance on climate change

Thematic analyses across SASSY segments found extreme segments were significantly more likely to respond that *Nothing* would change their current beliefs and give less *Unsure* responses. These findings support previous research showing the *Alarmed* and *Dismissive* have the firmest climate beliefs^21^ and their resistance to anything that would change their mind is consistent with their high attitudinal certainty^8^. Past research has linked confidence in and rigid adherence to beliefs with a reduced propensity to seek new information^44^ and a tendency to selectively pursue confirmatory information^45^. *Cautious* and *Concerned* participants were relatively less likely to state that *Nothing* would change their mind, which suggests reduced dogmatism and a greater likelihood of being persuaded by climate communications. Given the *Cautious* and *Concerned* have lower issue engagement, though general acceptance of climate change^46^, communications that increase issue engagement may be particularly fruitful. Recommendations for this group therefore include interventions that make climate change more personally relevant, such as by linking it to shared public health concerns^24,25^ or using persuasive narratives^47^.

*Evidence and Information* was the most cited theme across segments (especially the *Cautious*) but less so for the *Dismissive*. Participants particularly emphasized “scientific” and “statistical” evidence. The prevalence of this subtheme, despite a vast body of research, highlights the importance of effectively communicating existing empirical evidence supporting anthropogenic climate change. More than a quarter of responses coded to *Evidence and Information* also called for simpler or clearer climate change explanations. For example, one *Cautious* participant wrote, “I’m not sure if people are overreacting or not. It needs to be scientifically explained but in plain [E]nglish what is happening”. Such responses are consistent with the lower issue involvement of the *Cautious*, who may be less willing to engage in effortful information processing^8^. This finding suggests opportunities to facilitate interpretation of climate science by improving education and research translation.

It is unlikely, however, that scientific evidence alone would successfully shift the opinions of climate change deniers. *Dismissive* and *Doubtful* segments are instead more likely to argue against such evidence in motivated defense of their current positions^8^. This tendency could be seen in responses from skeptical segments that expressed a lack of trust in current empirical evidence. Relative to other groups, these segments more often mentioned the need for “unbiased” or “transparent” information. For example, one *Dismissive* participant responded, “real fact[s] not hysteria lies and false modelling”, implying fraud in climate change research. Previous research investigating the belief-updating tendencies of climate audience segments similarly found resistance among disbelieving segments. Andreotta and colleagues^48^ identified three segments (*Acceptors*, *Fencesitters*, and *Sceptics*) based on beliefs about climate change and its causes, consequences, and solutions. Exposing these audiences to scientific information allowed researchers to examine their propensity to update beliefs. *Sceptics* were the most resistant to updating their beliefs in response to scientific evidence. *Acceptors* more readily updated their estimates but already supported climate policies. The researchers thus recommended targeting communications towards *Fencesitters*, who also updated their beliefs but may be vulnerable to mis- or dis-information.

The *Dismissive* and *Doubtful* segments’ frequent references to definitive or irrefutable “proof” further challenge the idea that clearer explanations of climate science would effectively persuade them about the reality and risks of climate change. These segments instead seemed to require a higher level of evidence. Since science can never deliver absolute certainty, these responses reflect the establishment of “impossible expectations” about what research can show, which has been identified as a common technique used in science denial^49^. Direct or vicarious experiences of climate impacts may therefore be more effective than scientific evidence in shifting skeptical segments’ views^50^. A recent meta-analysis found that anecdotal evidence is more persuasive than statistics when issue involvement is high^51^. However, sharing personal stories of climate change harms can also motivate issue involvement in skeptical audiences by increasing worry and compassion^52^. Linking climate impacts to issues and objects of concern may thus be an effective communication strategy for climate denialists^53^. Indeed, almost a third of *Evidence and Information* responses by skeptical segments claimed “personal” experiences of climate change could shift current beliefs. However, one meta-analysis found little association between experiencing climate change and people’s views on the issue^54^, which casts doubt on experiential evidence’s effectiveness. The relatively high proportion of *Doubtful* responses that cited observable or experiential evidence nonetheless suggests this approach’s effectiveness could be further tested for this segment.

Beyond types of evidence and information, our analyses underscored the importance of *Trusted Sources*, particularly for the *Alarmed* (and less so for the *Doubtful*). When scientists are trusted, people are more likely to adopt scientific conclusions^55^. Subtheme analyses found that scientists and experts were the most cited trusted sources across segments. However, in line with previous research^56^, while climate change believers generally held positive attitudes towards these figures, skeptics were more prone to doubt their claims. The *Disengaged*, *Doubtful*, and *Dismissive* generally expressed greater distrust towards existing sources of information and a desire for unbiased and “qualified” experts. Some responses even referred to climate change as a “hoax” or “gee-up” created by self-serving actors. Such responses appear to reflect science-related populist attitudes, which challenge the expertise and authority of scientists and instead attach greater importance to the common sense of “everyday people”^57^. This lack of trust suggests an urgent need to restore public faith in those that produce and disseminate climate research and highlights the potential value of engaging ingroup influencers who may benefit from increased perceptions of trust and credibility^58^. Indeed, Leiserowitz and colleagues^8^ found that the *Doubtful* and *Dismissive* placed more trust in their family and friends for climate change information, while the *Alarmed* and *Concerned* placed trust in scientists and scientific organizations.

Across all segments except the *Disengaged*, many participants also cited a need for consensus, particularly among scientists. For example, one *Doubtful* participant wrote, “When the science is in, too many conflicting views and data in the scientific world.” Such responses reflect the known value of communicating the current scientific consensus about anthropogenic climate change^12^. Previous research suggests consensus messaging may increase not only belief in human-induced climate change but also support for mitigation policies^59^ and climate action for people who identify as liberal^11^. However, evidence also indicates such messaging may have rebound effects, reducing perceived consensus, acceptance of anthropogenic climate change, and climate policy support for certain groups^60^. Future research should further investigate how and why the effectiveness of consensus messaging, and trust in scientists more broadly, differs across climate opinion segments.

The *Action* theme was most prevalent among the *Alarmed* and *Concerned*. Subtheme analyses across these segments, as well as the *Cautious*, indicated that actions by government and corporations and widespread individual behavior change would signal climate change’s severity. The prevalence of these subthemes reflects the general perception that radical government action and individual pro-environmental behavior is costly^61^. Effectively communicating such efforts could highlight climate change’s importance and urgency, thus strengthening pro-climate attitudes. These initiatives could be bolstered by normative messaging about others’ behavior to further shift attitudes and actions^62^ and reduce misperceptions about others’ stances on climate change^63^. In addition to strengthening existing beliefs, these segments suggested *Action* would provide optimism and hope. For example, an *Alarmed* participant wrote, “If I saw meaningful government action on climate change I might not be as pessimistic about it.” Such responses highlight the importance of supporting climate advocates, whose needs are often sidelined by campaigns and interventions targeting fence-sitters and skeptics^64,65^.

The *Disengaged*, *Doubtful*, and *Dismissive’s* responses were comparatively less likely to mention *Action*. However, action on a global scale was a more common subtheme across these segments, with many respondents suggesting Australia’s pro-climate efforts would be negated by high emissions elsewhere (e.g., China and India). Communication efforts could respond to these views by emphasizing a socially-just transition or Australia’s high per capita emissions. However, the persuasiveness of such values-based messaging would likely depend on individuals’ ideological and moral beliefs^66^. The importance of framing messages to appeal to all groups in society was further highlighted in references to depoliticization and deradicalization across *Dismissive* responses. For example, one participant responded, “[…] climate change advocacy has become an intolerant, ideologically driven dogma […] rational analysis or even civil discourse has become largely impossible.” Such responses underscore the urgent need for bipartisan discourse and action that signals consensus about climate change, and further highlight the potential value of leveraging ingroup messengers and personal connections^67^.

Overall, our thematic analyses reveal important insights about what factors individuals believe could shift their views on climate change. However, considerable effort has already been expended in initiatives addressing many participant concerns, to modest effect (e.g.,^68^). This raises the question: is there a difference between what individuals *believe* would change their minds about climate change and what shifts their beliefs *in practice*? Moreover, it is possible our question elicited something approximating a cultural worldview of climate change^69^, rather than an expression of participants’ belief-updating tendencies. From this perspective, participants’ responses may be considered discursive tools, or rhetorical arguments, adopted to justify their attitude towards a contested issue. Evidence nevertheless supports the effectiveness of many of the participant-generated insights in changing climate views (e.g., scientific consensus and communication by trusted sources). However, novel ideas ought to be treated with appropriate skepticism and empirically tested before forming firm recommendations for climate change communications. Future research endeavors must also contend with questions about whether and how to allocate efforts to change the views of those stubbornly against the very idea of climate change, versus motivating those who are attitudinally aligned to engage in pro-climate behaviors.

### RQ 2: GPT as a tool for thematic analyses

Our research demonstrates GPT is an effective and efficient tool for managing the abundant data emerging in the social sciences. GPT performed highly accurate thematic analyses, comparable to human performance in identifying the presence or absence of themes. GPT achieved “almost perfect” agreement with both human coders for all themes, reaching levels typically accepted in content analysis research with only human coders^41,42^. Additionally, GPT-4o was highly cost-effective and time-saving: data was processed in a few hours, costing less than US$0.01 per response. These metrics are favorable compared to the costs associated with employing human coders.

Despite its high accuracy, GPT sometimes misfired when identifying themes in responses that failed to directly answer the question asked to participants, particularly for the *Action* theme. For example, GPT coded the following response as *Action*, although the behaviors described do not directly address what would change the participant’s mind: “I think everyone should be mindful of everything they buy and use, to stop waste going into the natural environment.” We discuss other common instances of GPT mis-categorization in the Supplementary Materials. Overall, however, our findings support GPT’s effectiveness and efficiency in handling thematic coding for large amounts of text data and add to growing evidence demonstrating LMMs’ capacity to perform a variety of tasks at human or near-human levels^35,70–73^. It is worth noting that the use of LMMs, such as GPT, involves environmental costs, which researchers should consider as part of the broader ethical considerations associated with computational methodologies.

### Limitations and future directions

These findings pave the path for important future work. We assessed what factors participants believe would change their mind about climate change, achieving participant-informed approaches to opinion change. However, it is unclear whether participants interpreted our question in a consistent manner (e.g., whether *Alarmed* participants universally described what would make them doubt climate change). Additionally, we cannot say whether the factors described by participants would change current opinions in practice. People may have poor insight into what influences their thoughts and actions, and, for example, often underestimate the influence of others’ actions on their behavior^74^. Future work should assess the accuracy of people’s beliefs about what would motivate climate opinion change (e.g., through interventions targeting these themes across different segments). Our goal was a higher-level thematic analysis of participant responses. This approach was necessary to interpret the copious data analyzed but may have resulted in loss of detail. For example, extracting common themes across SASSY segments prevented examination of unique themes for specific segments. Future work may wish to conduct in-depth analyses of a subset of responses to uncover potentially novel themes. We also note the current sample had higher educational attainment relative to the Australian population, and education level predicted the presence or absence of themes. Though effects were small, we advise future researchers to be mindful of potential demographic effects when extrapolating to larger populations. Finally, while GPT achieved “almost perfect” intercoder reliability with both human coders, agreement rates were lower than between the two human coders. Future research should continue exploring LLMs’ potential in qualitative research as more advanced models emerge.

## Conclusion

Climate change is thought to be the most catastrophic existential crisis facing the world^75^. Now more than ever, we must understand public views about the existence and causes of climate change and what might shift opinions towards both skeptical and accepting orientations. Our analyses revealed important insights about what it would take to change Australians’ current climate beliefs, including opinion-change resistance in extreme segments, the universal importance of evidence and information and trusted sources, and the value of framing climate action in a way that resonates with different audiences. We also demonstrated GPT API’s efficacy in identifying themes from qualitative text in a large, representative dataset. GPT exceeded minimum reliability standards, achieving “almost perfect” agreement with two human coders. Given climate change’s rapid progression, leveraging AI in psychological research to analyze large datasets in a time- and cost-efficient manner offers significant opportunities to enhance our understanding of human behavior and develop strategies to mitigate this crisis.

## Materials and methods

### Participants and procedure

We collected data from 5110 participants between August and September 2021 through Qualtrics Panels, which recruits a population-matched sample along age, gender, and location demographics. Participant ages ranged from 18 to 89 years (*M* = 47.21, *SD* = 17.68), and 50.9% identified as women, 48.8% as men, and 0.3% preferred another term or preferred not to answer. Most participants (65.0%) reported living in a capital city, followed by a regional town (22.1%) or rural area (11.9%), with 0.9% specifying another area or not answering the location question. The sample had a median educational attainment of Advanced diploma/Associate degree level, and a median annual income of AUD$ 31,200 − 41,599. Supplementary Table S7 shows that the sample demographics closely matched the Australian population in 2021 across age, gender, and geographical location, although the sample had higher levels of educational attainment (46.4% had achieved a bachelor’s degree or higher, compared with 26.1% of the population; see Supplementary Materials for full comparisons). The Australian National University Human Research Ethics Committee approved ethical aspects of the research (#2020 − 429), and all methods were carried out in accordance with the relevant regulations and guidelines. Informed consent was inferred from all participants by their survey completion after reading a plain language information sheet.

### Measures

Survey questions and wording are shown in full in Supplementary Table S8 in the order they were presented to participants.

#### Climate change attitude profile

Chryst et al.’s Six Americas Short Survey (SASSY)^21^ enables audience segmentation across six attitudinal profiles^46^ with just four items. Participants indicated how important climate change or global warming was to them, along a 5-point scale from “not at all” to “extremely important”. They reported how worried they were along a 4-point scale from “not at all worried” to “very worried”. They also rated how much they thought climate change or global warming would harm them personally and future generations from five response options (“not at all” to “a great deal”, and with the option to say “don’t know”). As we report elsewhere^9^, 5104 participants completed all four items and we could thus examine their profile membership. Participants were randomly assigned to complete the four SASSY questions under different rating contexts. Specifically, half of participants responded to these questions about “*climate change*”, and the remainder answered about “*global warming*”. This manipulation was included to test whether participants responded differently to the issue depending on the label used, which is reported on elsewhere. As labelling had no significant effect on the proportion allocated to each segment, meaning participants responded in similar ways regardless of the label they were exposed to (see^9^), for the purposes of this study, we collapsed across rating conditions.

Although the Six Americas profiling tool was developed using US samples^46^, it has been used with Australian samples in both long-form and the SASSY tool^22,76^. An independent, inductive, data-driven latent class analysis using Australian data and the original set of questions from the Global Warming’s Six America’s survey identified the same six segments found in the US and separated by their attitudinal valence and issue involvement^22^, which suggests the segmentation approach is valid for use in Australia. Chryst et al.^21^ validated the SASSY tool and found that the four items retained from the original longer questionnaire in the United States performed well at assigning respondents to audience segments. The SASSY tool has since been used to classify Australian respondents into segments^9,20^.

That said, alternative segmentation approaches, such as those relying on a different set of questions to the ones incorporated in the Six Americas or SASSY questionnaires, have identified a narrower set of audience segments^77^. Indeed, the number and composition of audience segments varies based on the items used in the segmentation analysis (see^9^ for a discussion). An alternative approach would have been to use data-driven methods to segment our sample in search of a novel set of audience segments. We prioritized identifying novel themes in open-text responses and thus elected to use the validated SASSY tool to derive established and externally validated audience profiles. However, future research could build on our work to use data-driven methods to also identify climate opinion groups.

#### What would change your mind?

Directly after completing the SASSY and a range of other climate belief measures, participants were asked: “Please think now about your opinions on climate change, whether you accept the climate is changing or not, whether human activity is causing climate change or not, how strong and certain those opinions are, and so on. We are interested in what might change your opinions. In your own words, can you please describe what might change your opinions about climate change from how they are now?” Participants typed their response in an open text box. In total, 4857 participants responded to the question, and GPT was able to assign 4849 to a theme.

### Data analysis

#### Identifying themes and subthemes

We developed a coding framework by conducting inductive content analysis on a random sample of 322 responses to the open-ended question (6.6% of responses to this question). Two coders independently identified all themes expressed in the data, then collectively negotiated the most frequently identified themes across responses. The final codebook consisted of six themes (see Table 1), which were found across all SASSY segments. Although a larger number of themes may have captured greater complexity and nuance, this approach would likely come at the cost of decreased reliability for both the human coders and GPT^78,79^. The human coders and GPT used the codebook to independently analyze a random sample of 400 responses (8.2% of responses), including the 322 responses initially analyzed to develop the codebook. We noted multiple themes for responses that featured equally dominant themes (see Supplementary Table S2).

After sufficient intercoder reliability was established and GPT thematically coded all participant responses (detailed below), the two human coders conducted another round of inductive content analysis to identify the dominant subthemes for the *Evidence and Information*, *Trusted Sources*, and *Action* themes. The coders independently identified all subthemes expressed in responses coded to the three substantive themes, then collectively negotiated the most frequently identified subthemes across responses. Between five to seven subthemes were identified for each theme (see Table 1).

#### GPT thematic coding

We first used OpenAI’s GPT to identify the presence or absence of the themes assessed by human coders in the previous step. There are several GPT models that can be used to complete thematic analyses. While GPT-3.5 is both fast and very low-cost, it tends to produce inaccurate results relative to more advanced models such as GPT-4. For example, while GPT-3.5 struggles to engage in inductive reasoning (property induction i.e., inferring that unknown objects share certain properties with known objects based on observed similarities), GPT-4.0 performs at human-level across most task variations^80^. To enhance accuracy, we used one of the more advanced models available to the public as of January 2025: *gpt-4o*. Using the OpenAI API, we repeatedly prompted GPT to identify the presence or absence of themes in participants’ qualitative responses using R following previous methods^31^. GPT was specifically given the following prompt:

“I want you to label these responses with themes that correspond to those responses, labelling each response with a number ONLY - ‘1’ for Nothing, ‘2’ for Evidence and information, ‘3’ for Trusted sources, ‘4’ for Action, ‘5’ for Unsure, and ‘6’ for N/A. I only want the number associated with the theme as the output. If responses refer to multiple categories, then all relevant categories should be assigned to that response. For example, if a response refers to both ‘1’ for Nothing and ‘2’ for Evidence and information, the output should be written in the following format: ‘1,*2’.*

These responses are answers to the following question: ‘Please think now about your opinions on climate change, whether you accept the climate is changing or not, whether human activity is causing climate change or not, how strong and certain those opinions are, and so on. We are interested in what might change your opinions. In your own words, can you please describe what might change your opinions about climate change from how they are now?’

Here are the definitions of the six themes that I want you to use:...”

GPT was then provided with the six definitions presented in Supplementary Table S1. This process was first completed for the 400 participant responses assessed by human coders. After sufficient reliability was established in these initial responses (described below), GPT assigned themes for all remaining participant responses. This process was then repeated to identify the presence or absence of subthemes across responses coded to *Evidence and Information*, *Trusted Sources*, and *Action* (see Table 1). See OSF (https://osf.io/4hjy8/) for R code for accessing the GPT API.

#### Assessing GPT reliability

 We analyzed intercoder reliability for each theme to determine whether it would be appropriate to use GPT to thematically code participant responses (see Table 3). We conducted reliability analysis in R using the irrCAC library^39^ (see https://osf.io/4hjy8/ for R code). Given “almost perfect” agreement was achieved between GPT and both human coders for each theme^40^, we determined it would be appropriate to use GPT to code all responses for major themes and subthemes.

#### Analyzing the prevalence of themes across segments

 We ran the responses to the four SASSY items through the online SASSY tool (https://climatecommunication.yale.edu/visualizations-data/sassy) to derive each participant’s audience segment (see^21^ for a full discussion of this method). The prevalence of each segment is reported in the Results section.

We then used a series of Chi-square tests to examine how frequently each theme was observed in each of the six audience profiles. A significant Chi-square test indicates that in at least one cell within the contingency table, there is a disproportionate number of people using a given theme, but it cannot locate the specific source of the misfit between observed and expected frequencies. To do this, we followed up on significant overall Chi-square tests using Beasley and Schumacker’s post hoc analysis^81^, which compares observed to expected frequencies in each cell to locate which cells in the contingency table are significantly different from that expected by chance. Specifically, this analysis examines adjusted standardized residuals in each cell, which are akin to z-scores and thus values greater than +/-1.96, corresponding to a *p*-value less than 0.05. For each analysis, we divide the critical alpha value by 12 because we are essentially conducting 12 post-hoc analyses (0.05/12 = 0.004). To obtain *p*-values for each cell, we transformed the adjusted residuals into Chi-square values, and derived *p*-values at 1 degree of freedom.

#### Analyzing the prevalence of subthemes across segments

 We analyzed the prevalence of subthemes across responses by each SASSY segment that were coded to *Evidence and Information*, *Trusted Sources*, and *Action.* However, we could not test for statistically significant differences because of insufficient power. We did not include *Nothing* or *Unsure* responses in these analyses because responses coded to these themes were highly homogeneous and did not feature substantive subthemes.

## Electronic supplementary material

Below is the link to the electronic supplementary material.

Supplementary Material 1

## Data Availability

Data and analysis code are available on the Open Science Framework: https://osf.io/4hjy8/.
