# Supplementary material for: A thematic analysis of what Australians state would change their minds on climate change
Source: Sci Rep. 2025 Apr 22;15:12989. doi: 10.1038/s41598-025-96714-z (PMC12015523; doi:10.1038/s41598-025-96714-z)
Supplement: Supplementary file 1 — Supplementary Material 1 [file 41598_2025_96714_MOESM1_ESM.docx]

**A thematic analysis of what Australians state would change their minds on climate change**

Amy S G Lee, Kelly Kirkland, Samantha K Stanley, Abby Robinson, Zoe Leviston, and Iain Walker

**Supplementary Materials**

**Full Definitions of Themes**

We present the exact definitions used to feed themes and subthemes to GPT in Table S1.

*Table S1. Full definitions of dominant themes and subthemes.*

| **Themes** | **Subthemes** |
| --- | --- |
| 1. **Nothing:** This theme applies to respondents who express a strong commitment to their current views and state that nothing would change their mind. For example, those who say ‘none’ or ‘nothing’ are referring to nothing changing their mind. | Not applicable |
| 1. **Evidence and Information**: This theme applies to respondents who state that more evidence, information, and/or education would change their mind. This includes personal experience of or observable changes in weather patterns, scientific data or research, information about the role of human activity in climate change, greater education about climate change, and/or increased transparency. Respondents who simply express a desire for more ‘facts’, ‘data’, or ‘research’ without specifying what kind of evidence or information is required are still expressing a desire for increased evidence and information. | - 1. **Scientific evidence**: Respondent states a desire or need for scientific evidence or research.   2. **Anthropogenic climate change**: Respondent describes a need for evidence that climate change is or is not caused by humans.   3. **Observable/experiential evidence or personally feeling climate impacts**: Respondent describes personally experiencing or observing that climate change is or is not occurring.   4. **Unbiased or transparent information**: Respondent describes a desire for more transparent information or less biased information.   5. **Simple/clearer information or education**: Respondent describes a need for clearer or simpler information or explanations about climate change. Alternatively, respondent expresses a desire for more education.   6. **Legitimate information**: Respondent expresses a desire for better, proper, or more legitimate information.   7. **Definitive or irrefutable evidence**: Respondent expresses a desire for definitive, irrefutable, or absolute evidence/proof that the climate is or is not changing. |
| 1. **Trusted Sources**: Respondents who fall under this theme state that trusted sources of information would change their mind. Trusted sources of information could include experts, the scientific community, and/or public figures. Participants who state that greater consensus would change their mind should also be categorized as requiring trusted sources. | - 1. **Scientists & experts in the field**: Respondent describes opinions from scientific or environmental organizations, scientists, or experts in the field.   2. **Consensus**: Respondent comments on a need for greater consensus (e.g., scientific/expert consensus, public consensus, political consensus, consensus between different groups).   3. **Unbiased sources of information or no vested interests**: Respondents describe a need for unbiased sources of information or sources of information that do not have vested interests.   4. **Media**: Respondent comments on the role of the media. Comments could include expressions of distrust towards the media or a desire for more media coverage or news reports.   5. **Government**: Respondent comments on the government or on government bodies. Comments could include desire for information from government/government bodies or distrust towards government/government bodies.   6. **Distrust towards existing information sources**: Respondent expresses distrust for existing information produced by individuals and public or private institutions. These institutions can include scientists and scientific organizations, the government, the media, politicians, and other public figures. |
| 1. **Action**: Respondent states that actions taken by the government, corporations, or the public would change their mind. Respondents who state that a change in how people talk about or behave regarding climate change are also referring to greater action changing their minds. | - 1. **Action by government**: Respondent states that climate-related government actions (e.g., stronger climate policies) would change their mind about climate change.   2. **Actions by corporations and big business**: Respondent states that climate-related actions by corporations (e.g., stronger climate policies, pro-climate practices) would change their mind about climate change.   3. **Widespread individual behavior change**: Respondent states that actions by individuals, people, humans, or the general public could change their mind.   4. **Action on a global scale**: Respondent refers to actions by countries/governments around the world, beyond Australia.   5. **Depoliticization, deradicalization**: Respondent mentions a need for reduced politicization, radicalism, or extremism. Alternatively, respondent mentions a desire for increased level-headedness and rational debate between opposing parties. |
| 1. **Unsure**: Respondent states they are unsure what would change their mind. | Not applicable |
| 1. **N/A**: Respondent goes off on tangents that do not directly address the question: “What would change your mind about climate change?”. For example: “In this Covid situation I noticed that all human activities suddenly slow down, then we experienced a fresh climate again, clear river, no air pollution etc, so I think the climate change is the after effects of human inventions”, “Human impact affects climate change”, and “I feel like a lot of the information I hear is peoples opinions not facts” would all be considered N/A responses because they do not address the question. | Not applicable |

**Theme Co-occurrence**

Open-ended participant responses about what it would take to change their mind about climate change often featured more than one theme. We present the frequency of theme co-occurrence in Table S2.

*Table S2. Theme co-occurrence matrix*.

|  | Nothing | Evidence & Information | Trusted Sources | Action | Unsure | N/A |
| --- | --- | --- | --- | --- | --- | --- |
| Nothing | **1161** |  |  |  |  |  |
| Evidence & Information | 95 | **1675** |  |  |  |  |
| Trusted Sources | 26 | 308 | **455** |  |  |  |
| Action | 96 | 120 | 51 | **956** |  |  |
| Unsure | 2 | 24 | 8 | 6 | **372** |  |
| N/A | 38 | 12 | 4 | 7 | 10 | **977** |

*Note*. Frequency of each theme across the full sample is bolded in diagonal.

**“Not Applicable” Theme**

We instructed GPT to categorize responses into one of six themes and describe the content and prevalence of the five substantive themes in the main manuscript. The final theme was called “N/A”, and included responses that did not directly address the question (see Table S1 for full details). As shown in Table S3 and Figure S1, the N/A theme was used disproportionately more by *Doubtful* and *Dismissive* participants, and less by those in the *Concerned* segment. Table S4 shows “almost perfect agreement” between human coders, and between each human coder and GPT, in assigning responses to the N/A theme.

*Table S3. Contingency table showing the presence of N/A theme by SASSY segment.*

|  | N/A |
| --- | --- |
| Alarmed | 17.7 |
| Concerned | **17.4** |
| Cautious | 20.9 |
| Disengaged | 19.1 |
| Doubtful | **25.0** |
| Dismissive | **27.8** |
| Chi-square test | χ^2^(5) = 33.98, *p* < .001, V = .08 |

*Note.* N = 4844. V = Cramer’s V effect size. Bolded cells depict significant findings at the adjusted threshold *p*-value < .004. Shading is used to depict overrepresented (green) and underrepresented (orange) use of themes.

*Figure S1. Standardized proportion of major themes across SASSY segments (including N/A theme).*

*Table S4. Summary statistics for intercoder reliability (including N/A theme).*

|  | Coder 1 & 2 | | | | Coder 1 & GPT | | | | Coder 2 & GPT | | | | |
| --- | --- | --- | --- | --- | --- | --- | --- | --- | --- | --- | --- | --- | --- |
|  | Gwet’s AC(1) | 95% CI | | Percentage Agreement | Gwet’s AC(1) | 95% CI | | Percentage Agreement | Gwet’s AC(1) | 95% CI | | Percentage Agreement |  |
|  |  | *LL* | *UL* |  |  | *LL* | *UL* |  |  | *LL* | *UL* |  |  |
| Nothing | 0.96 | 0.94 | 0.99 | 98 | 0.96 | 0.93 | 0.98 | 97 | 0.95 | 0.93 | 0.98 | 97 |  |
| Evidence & Information | 0.95 | 0.92 | 0.98 | 97 | 0.87 | 0.82 | 0.92 | 93 | 0.87 | 0.83 | 0.92 | 93 |  |
| Trusted Sources | 0.97 | 0.95 | 0.99 | 97 | 0.94 | 0.91 | 0.97 | 95 | 0.95 | 0.93 | 0.97 | 96 |  |
| Action | 0.98 | 0.97 | 1.00 | 99 | 0.88 | 0.83 | 0.92 | 91 | 0.86 | 0.82 | 0.91 | 90 |  |
| Unsure | 0.99 | 0.98 | 1.00 | 99 | 0.98 | 0.97 | 1.00 | 99 | 0.98 | 0.96 | 1.00 | 98 |  |
| N/A | 0.93 | 0.90 | 0.96 | 96 | 0.86 | 0.81 | 0.90 | 90 | 0.83 | 0.78 | 0.88 | 89 |  |

*Note.* CI = confidence interval; *LL* = lower limit; *UL* = upper limit. All Gwet’s AC(1) values reflect “almost perfect agreement” (Landis & Koch, 1977).

**Post-hoc Analyses**

*Table S5. Z-scores and associated p-values to identify cells in the Chi-square contingency table that significantly differ from chance.*

|  | Nothing | | Evidence & Information | | Trusted Sources | | Action | | Unsure | | N/A | | |
| --- | --- | --- | --- | --- | --- | --- | --- | --- | --- | --- | --- | --- | --- |
|  | Adj. Res. | *p* | Adj. Res. | *p* | Adj. Res. | *p* | Adj. Res. | *p* | Adj. Res. | *p* | Adj. Res. | *p* |  |
| Alarmed | 3.343 | .001 | -1.811 | .070 | 3.575 | < .001 | 8.486 | < .001 | -7.164 | < .001 | -2.464 | .014 |  |
| Concerned | -3.665 | < .001 | 1.019 | .308 | -0.171 | .864 | 3.391 | .001 | 0.868 | .385 | -2.927 | .003 |  |
| Cautious | -7.313 | < .001 | 4.603 | < .001 | -0.487 | .626 | 0.410 | .682 | 1.505 | .132 | 0.645 | .519 |  |
| Disengaged | -2.588 | .010 | -0.259 | .796 | 1.052 | .293 | -3.717 | < .001 | 8.458 | < .001 | -0.340 | .734 |  |
| Doubtful | 2.314 | .021 | -1.295 | .195 | -3.203 | .001 | -8.243 | < .001 | 4.030 | < .001 | 3.386 | .001 |  |
| Dismissive | 11.012 | < .001 | -4.186 | < .001 | -1.280 | .201 | -7.011 | < .001 | -3.013 | .003 | 3.739 | < .001 |  |

*Note.* Adj. Res. = Adjusted standardised residual, equivalent to a z-score. These are equivalent in size (though opposite in sign direction) for present/absent categories and for simplicity are presented for theme present. We use the adjusted critical alpha value of *p* < .004 to determine statistical significance.

**Demographic Analyses**

A series of binary logistic regressions were run to assess the likelihood of the presence of each theme given participants’ sociodemographic responses. The sample size for these analyses began with the 4844 in the main manuscript who answered and could be coded to a theme and omitted those with missing demographic information. For the categorical variable of gender, we recoded responses to binary for inclusion as predictors in the regression, which omitted non-binary participants and those who preferred not to give their gender from these analyses. Thus, our sample size for these analyses was 4783.

We present odds ratios in Table S6 whereby values greater than one indicate increasing odds that the theme is present for a given demographic, and values lower than one indicate decreasing odds that the theme is present.

Our findings showed that men were less likely than women to be *Unsure* about what would change their mind. Each year of increased age was associated with a small increase in the likelihood of saying *Nothing* would change one’s mind or giving an irrelevant response (coded as *N/A)*, and a slight decrease in likelihood of the presence of *Evidence and Information, Trusted Sources* and *Unsure* themes in participant responses. Income was not associated with the presence of any themes, whereas education was a significant predictor of every theme: *Evidence and Information, Trusted Sources, Action*, and *N/A* were each more likely at higher education levels, and *Nothing* and *Unsure* were less likely with increased education. Increases in right-wing political orientation were associated with greater likelihood of responses coded as *Unsure* or *N/A* and lower likelihood of reporting *Nothing*, *Evidence and Information,* or *Trusted Sources* would change one’s mind. However, across all models, the demographic variables explained only a small portion of the variance in likelihood of reporting each theme. Specifically, the pseudo R^2^ values indicated that between 0.1-0.2% of variance was explained in the case of *Action*, and up to 3-5% of variance explained in *Nothing*.

*Table S6. Odds ratios for demographic variables predicting presence or absence of each theme.*

|  | Nothing  *Exp(B)* | Evidence & Information  *Exp(B)* | Trusted sources  *Exp(B)* | Action  *Exp(B)* | Unsure  *Exp(B)* | N/A  *Exp(B)* |
| --- | --- | --- | --- | --- | --- | --- |
| Gender (male as reference category) | 1.05 | 0.94 | 0.90 | 1.11 | 1.62*** | 1.06 |
| Age in years | 1.02*** | 0.98*** | 0.99** | 1.00 | 0.99** | 1.01*** |
| Income | 0.99 | 1.01 | 0.99 | 0.99 | 1.03 | .99 |
| Education | 0.93*** | 1.03** | 1.09*** | 1.03 | 0.89*** | 1.06*** |
| Right-wing political orientation | 0.99*** | 0.99*** | 0.99*** | 1.00 | 1.00 | 1.02*** |
| Pseudo R^2^ range^†^ | .03 - .05 | .03 - .05 | .02-.04 | .001-.002 | .01-.03 | .03-.04 |

Note. * *p* < .05, ** *p* < .01, *** *p* < .001. ^†^ Denotes Cox & Snell R^2^ and Nagelkerke R^2^ values, respectively. N = 4783.

*Table S7. Demographics of survey respondents compared with the Australian population.*

| Demographic | Category | All study participants (*N*= 5110) | Participants retained for demographic analyses  (*N*=4783) | Australian population in 2021 census* |
| --- | --- | --- | --- | --- |
| Age | 20-24 | 10.9% | 10.4% | 6.2% (8.2%) † |
|  | 25-34 | 17.2% | 16.9% | 14.3% (18.8%) |
|  | 35-44 | 17.0% | 16.8% | 13.7% (18.1%) |
|  | 45-54 | 15.1% | 15.2% | 12.7% (16.7%) |
|  | 55-64 | 16.6% | 17.0% | 12.1% (15.9%) |
|  | 65-74 | 15.3% | 15.8% | 9.7% (12.8%) |
|  | 75+ | 5.7% | 5.9% | 7.5% (9.9%) |
| Gender | Female | 50.9% | 50.2% | 50.7% |
|  | Male | 48.8% | 49.8% | 49.3% |
|  | Not otherwise classified | 0.3% | - | - |
| State / Territory | Australian Capital Territory | 1.7% | 1.8% | 1.8% |
|  | New South Wales | 31.1% | 31.0% | 31.8% |
|  | Northern Territory | 0.4% | 0.4% | 0.9% |
|  | Queensland | 19.1% | 19.0% | 20.3% |
|  | South Australia | 7.4% | 7.5% | 7.0% |
|  | Tasmania | 3.1% | 3.1% | 2.2% |
|  | Victoria | 26.2% | 25.9% | 25.6% |
|  | Western Australia | 10.8% | 11.0% | 10.5% |
| Education Level | Year 8 or below | 0.7% | 0.7% | 3.2% |
|  | Year 9 | 1.7% | 1.7% | 3.3% |
|  | Year 10 | 8.0% | 8.1% | 10.4% |
|  | Year 11 | 3.2% | 3.1% | 4.6% |
|  | Year 12 | 19.0% | 19.0% | 14.9% |
|  | Diploma level | 14.4% | 14.5% | 5.6% |
|  | Advanced diploma and associate degree level | 6.6% | 6.9% | 3.7% |
|  | Bachelor degree level | 26.4% | 26.3% | 17.4% |
|  | Graduate certificate level | 4.9% | 4.8% | 0.5% |
|  | Graduate diploma level | 4.1% | 3.9% | 1.7% |
|  | Master degree | 9.7% | 9.8% | 5.5% |
|  | Doctoral degree | 1.3% | 1.3% | 1.0% |

*Note.* * Source: data drawn from the Australian Bureau of Statistics (<https://abs.gov.au/census/find-census-data/quickstats/2021/AUS> and <https://www.abs.gov.au/statistics/people/education/education-and-training-census/latest-release#data-downloads>). Note that Census age categories include all Australians, including those under 18 years of age, while educational attainment includes all Australian aged 15 years and over. † Figures in parentheses represent the proportion of the Australian population in each age bracket excluding people aged under 20 years (24.1% of the population). As the personal income brackets used by the Australian Bureau of Statistics for the 2021 reporting period (and as income brackets for the study were based on the previous Bureau categories), we are unable to directly compare the personal income of study participants with Australian population data.

**Common Instances of GPT Mis-Categorization**

Despite achieving “almost perfect” agreement with both human coders across all themes, GPT at times misfired when processing open-ended responses that did not directly address the question posed to participants. That is, GPT appeared to have some difficulty identifying and/or filtering out information that was irrelevant to what would change participants’ minds about climate change. As shown in Table 2 in the main manuscript, this tendency was especially apparent for the *Action* theme, for which GPT achieved a relatively lower percentage agreement with the two human coders (91% and 90%, respectively). Vague responses that described a need for action or pro-environmental behaviors without directly linking them to opinion change were a common source of error. For example, GPT categorized the following responses to *Action* rather than *N/A*, even though they did not directly address what would change participants’ current opinions about climate change:

1. “i accept climate change and something needs to be done now”
2. “its a problem we need to fix it”
3. “No using plastic, recycle more, clean the sea, watch out for koalas”.
4. “It's just a natural event of the world. People need to do the right thing, dispose of harmful chemicals/etc in the right places, not build on flood plains and they should be able to have a fire break around their house and be ale [sic] to log their property if they have to.”

The high co-occurrence of the *Evidence and Information* and *Trusted Sources* themes shown in Table S2 also suggests that GPT may have, at times, struggled to distinguish between these themes. We noted two common instances where GPT reacted to ambiguity in participants’ responses by coding them to both themes. First, GPT coded responses that described “peer reviewed” evidence to both *Evidence and Information* and *Trusted Sources*, even when responses appeared to focus more on the nature of the evidence than the messenger delivering it (e.g., "The only thing that could change my mind is peer reviewed, evidence based science” or “If peer reviewed scientific evidence can be presented that proves that climate change is not happening and not due to humans, I would have to consider it”). GPT also coded responses that appeared to describe “science” as an entity to both themes (e.g., “A change in the science- I trust it in the aggregate so if that found something new I would trust that” or “i feel that scinece [sic] can demonstrate clearly the evidence one way or the other. We follow science in terms of how we deal with covid. Why arent we following science with climate change action?"). It is important to note, however, that these types of ambiguities also led to inconsistencies between the two human coders and were debated during codebook development.

**Survey Questions**

Survey questions are detailed in Table S8 in the wording and order presented to participants. Note that these questions were embedded within a larger survey.

*Table S8. Wording and order of survey questions.*

| Variable | Question and response wording |
| --- | --- |
| Age | What is your age in years? Enter as a number |
| Gender | What is your gender?   - Male - Female - Other - Prefer not to say |
| Income | What is your total personal income per week (or per year), before any deductions?   - Negative income - Nil income - $1-$199 ($1-$10,399) - $200-$299 ($10,400-$15,599) - $300-$399 ($15,600-$20,799) - $400-$599 ($20,800-$31,199) - $600-$799 ($31,200-$41,599) - $800-$999 ($41,600-$51,999) - $1,000-$1,249 ($52,000-$64,999) - $1,250-$1,499 ($65,000-$77,999) - $1,500-$1,999 ($78,000-$103,999) - $2,000-$2,999 ($104,000-$155,999) - $3,000 or more ($156,000 or more) |
| Education | What is your highest level of educational attainment?   - Year 8 or below - Year 9 - Year 10 - Year 11 - Year 12 - Diploma level - Advanced diploma and Associates degree level - Bachelor degree level - Graduate certificate level - Graduate diploma level - Master degree - Doctoral degree |
| Location | How would you describe where you live?   - Capital city - Regional town - Rural area - Other – please specify: |
| Political ideology | Move the cursor below to the place on the slide which best represents your political views.   \|  \| \| \| --- \| --- \| \| Left-wing \| Right-wing \| |
| Six Americas | How much do you think climate change (global warming) will harm future generations of people?   - Don’t know - Not at all - Only a little - A moderate amount - A great deal   How important is the issue of climate change (global warming) to you personally?   - Not at all important - Not too important - Somewhat important - Very important - Extremely important   How worried are you about climate change (global warming)?   - Not at all worried - Not very worried - Somewhat worried - Very worried   How much do you think climate change (global warming) will harm you personally?   - Don’t know - Not at all - Only a little - A moderate amount - A great deal |
| What would change your mind? | Please think now about your opinions on climate change, whether you accept the climate is changing or not, whether human activity is causing climate change or not, how strong and certain those opinions are, and so on. We are interested in what might change your opinions.  In your own words, can you please describe what might change your opinions about climate change from how they are now?   \|  \| \| --- \| \|  \| \|  \| \|  \| |
